# Exclusive Breastfeeding and Other Foods in the First Six Months of Life: Effects on Nutritional Status and Body Composition of Brazilian Children

**DOI:** 10.1100/2012/468581

**Published:** 2012-10-21

**Authors:** Taís C. A. Magalhães, Sarah A. Vieira, Silvia E. Priore, Andréia Q. Ribeiro, Joel A. Lamounier, Sylvia C. C. Franceschini, Luciana F. R. Sant'Ana

**Affiliations:** ^1^Center of Biological Sciences and Health, Department of Nutrition and Health, Federal University of Viçosa, University Campus, Avenida P. H. Rolfs s/n, 36570-000 Viçosa, MG, Brazil; ^2^Center of Health Sciences, Department of Medicine, Federal University of São João Del-Rei, 36307-352 São João Del-Rei, MG, Brazil

## Abstract

*Objective*. To evaluate the effect of exclusive breastfeeding and consumption of other foods in the first six months of life in the nutritional status and body composition of children. *Methods*. A retrospective cohort study with 185 children aged from 4 to 7 years was monitored during the first months of life in a program of support to breastfeeding. We evaluated weight, height, waist circumference, and body composition by using DEXA. The nutritional status was assessed by the BMI/age index. The parameters of adiposity were classified by using as the cutoff point, the 85th percentile of the sample itself, according to gender and age. Confounding factors considered were variables related to maternal, pregnancy, birth, sociodemographic, health, lifestyle, and diet. Bivariate and multivariate analyses were performed, the latter by means of multiple logistic regression. *Results*. The median exclusive breastfeeding was 3 months. Of the children, 42.7% received cow's milk and 35.7% received infant formula. Regarding nutritional status, 21.1% of the children showed changes. The variables of infant feeding were not independently associated with nutritional status and body composition of the children and there were no differences between the groups studied. *Conclusion*. Breastfeeding was not a protective factor to overweight and body fat in children.

## 1. Introduction

The nutrition transition, characterized by a decrease in the prevalence of nutritional deficits and increased rates of overweight, obesity, and related diseases, has been occurring worldwide [1, 2] and has been described in all population groups, including in children [3].

In Brazil, assessments of the prevalence of growth deficits in preliminary comparisons of National Research on Demography and Health (PNDS) of 1996 and 2006 indicated a decrease of about 50% in the prevalence of malnutrition in childhood [4]. According to Brazilian data from the Research on Family Budgets (POF) held in 2008/2009, it was found that 33.5% of the population with ages between 5 and 9 years were overweight and 14.3% were diagnosed as obese [5].

Significant changes are observed in children with excess weight and body fat: components of metabolic syndrome and risk factors for cardiovascular disease [6–8], psychological and psychosocial problems [9]. In addition to health changes during childhood, it is noted that obese children tend to be obese adults [10]. It has already been reported that about one-third of preschool children and half of the school children who are obese keep this nutritional status when they become adults [11]. 

Given the perception of changes in nutritional status and health ever earlier, public health interventions of a preventive nature are important and should also occur in the early stages of development [12, 13]. 

It is suggested that exposure to environmental factors during critical periods such as during fetal life, childhood, and adolescence can influence the individual susceptibility to disease throughout life [14, 15].

With regard to childhood as a critical period of development, breastfeeding is more than what has been mentioned as a protective factor throughout life [16, 17]. In addition to the nutritional composition suitable for the child's development [18], breast milk would act upon behavioral aspects of mother-child relationship, the formation of the child's eating habits, and metabolic imprinting mechanism, due to its nutritional composition, presence of bioactive substances and hormones, resulting in protection to overweight and body fat, as well as cardiovascular diseases [16, 17]. 

Despite the evidence and hypotheses demonstrated, the effect of breastfeeding on the nutritional status and body composition is still controversial [19, 20]. Epidemiologic studies and meta-analyses have confirmed these results and found protection effect in the course of life associated to this practice [21, 22], but this has not been observed in all studies [23, 24]. The major discussions about the topic relate to publication bias, the need for control by confounding factors and form to obtain data of breastfeeding [19, 24–26].

 Through the aspects presented, this study aimed at evaluating the effect of exclusive breastfeeding and consumption of other foods in the first six months of life in the nutritional status and body composition of children from 4 to 7 years old participating in a project of extension supporting breastfeeding in the municipality of Viçosa, MG, Brazil.

## 2. Material and Methods

This is a retrospective cohort study [27] whose sample consisted of children aged between 4 and 7 years, monitored for the first months of life by a support program to breastfeeding (PROLAC) in the city of Viçosa, state of Minas Gerais, southeast Brazil, population around 72,220 inhabitants [28].

PROLAC is a program of the Federal University of Viçosa (UFV), whose main activities are the implementation of guidelines in the postpartum period with a view to promote breastfeeding, in partnership with the Human Milk Bank of the municipality of Viçosa and nutritional care to nursing mothers and children during their first year of life. The program began activities in August 2003 and has established protocols for the care and medical records to register the information and assessments, attending in PROLAC students of Nutrition of the Federal University of Viçosa, from the sixth period of the course and participated for at least 6 months of training to perform the activities.

Inclusion criteria for the initial stage of the study considered the following: perform nutritional monitoring for at least 6 months in the Program for children who received breast milk and for at least two months, provided that no mother's milk was offered to the children at any time during this period to children who had been fed with artificial milk or who had been weaned during followup at PROLAC, stillborn [29], not having been born with low weight or macrosomia [30], and presence of identification data in PROLAC's charts that allowed their residence location. The initial sample consisted of 256 children.

Three attempts of location were made. Additional inclusion criteria after the location of the infant were the written consent of parents or guardians and conducting all phases of the study. It was considered as exclusion criteria the presence of diseases, changes in health, or use of medication by the children that could interfere in their nutritional status or body composition.

Data collection was divided into two stages: retrospective, after consultation with the medical records of PROLAC (data relative to the 2003 to 2006), and data relating to children at ages evaluated in the study (years 2010 and 2011). The collection of retrospective data was performed by a single nutritionist, responsible for the investigation, with previous knowledge of the Program's routine. We obtained maternal and gestational data (prepregnancy BMI, gestational weight gain, and mother's smoking during pregnancy) and at birth (birth weight) evaluated as possible factors associated with nutritional status and body composition at later ages. The maternal prepregnancy BMI and gestational weight gain were evaluated according to reference of the medicine institute [31]. The birth weight was evaluated in three growing categories, with the first category representing children born with insufficient weight [32]. 

With respect to infant feeding, data were obtained from medical records on the practice of exclusive breastfeeding (EBF), consumption of cow's milk, infant formula, and age of introduction of solid foods in infant feeding. Exclusive breastfeeding (EBF) was evaluated as the type of practice in which the infant receives only breast milk, straight from the breast or expressed, or breast milk from another source, no other liquids or solids, except for drops or syrups containing vitamins, oral rehydration salts, mineral supplements or drugs [18].

Children aged between 4 and 7 years were evaluated for weight, height, waist circumference, and percent body fat (total body and regional android representing the abdominal fat). Weight was measured on a digital electronic scale, with a maximum capacity of 150 kg and sensitivity of 50 g. Height was measured using a vertical stadiometer attached to the wall, with a length of 2 meters, divided into centimeters and subdivided into millimeters. We adopted the techniques proposed by Jelliffe [33].

The nutritional status of the children was evaluated according to sex and age, using the anthropometric indices of weight/age (W/A), height/age (H/A), and Body Mass Index/age (BMI/A), classified according to anthropometric references of the World Health Organization (WHO) [34, 35]. For the calculations of the indices, the Software WHO Anthro Plus [36] was used and the diagnosis of the nutritional status was performed by following the recommendation in *z*-score of WHO [37]. For the evaluation of the EBF time effect and consumption of other foods in the nutritional status, the index used was the BMI/A and the *Z*-score >+1 was considered as changed.

The children's body composition was assessed using the equipment DEXA (Dual Energy X-ray absorptiometry). The variables considered were total body fat mass in grams, total body fat percentage, fat mass in grams, in the android region in grams and fat percentage in the android region. The total body fat percentage and android region fat percentage variables were categorized using as a cutoff the 85th percentile distribution of the sample by gender and age.

To measure waist circumference a tape measure was used, with a length of 2 meters, flexible, inelastic, divided into centimeters and subdivided into millimeters at the level of the umbilicus scar [38]. The measures were made in triplicate, being considered the two closest ones for the calculation of the average. The cutoff for categorization of the variable was the 85th percentile, obtained in the sample itself, specific for age and sex.

Possible confounding factors associated with nutritional status and body composition at the stage of life of children regarding the evaluations were obtained by applying questionnaires to mothers or guardians. The variables evaluated were sociodemographic and health, lifestyles, and diet. The habits of life were obtained using a questionnaire adapted from Andaki [39]. 

The food variables were obtained from three food records, completed on nonconsecutive days, including a weekend day [30] by the mother or guardian for the child's diet, supplemented by information in the school or daycare. Information on the frequency of consumption of fatty foods was obtained through a questionnaire of frequency as to food consumption prepared by the investigators. 

The analyses relating to food records were performed using the software DietPro 5.1 [40]. We evaluated the percentage of energy derived from lipids and carbohydrates and considered values above the upper limit of the Acceptable Macronutrient Distribution Range (AMDR) as increased [41]. 

The mean energy intake (Kcal) of three food records of each child was compared to its energy needs for the determination of the variable of energy balance. We calculated the Estimated Energy Requirement (EER), using the physical activity level (PAL) [30, 41], estimated according to the questionnaire on lifestyle previously reported. PAL factors used were those of mild and moderate activities (for children who practiced sports in addition to usual activities). The standard deviation of the energy requirement was considered 58 kcal for males and 68 kcal for females [30]. The cases in which the difference between the mean energy intake and the value of EER were above two standard deviations of the need [30, 42] were considered as positive energy balance. 

With regard to ethical aspects, the study was approved by the Ethics Committee on Human Research of the Federal University of Viçosa. The children were only included in the study by signing the consent form and all had returned nutritional consultation and, where necessary, forwarding of the consultation with a pediatrician. 

## 3. Statistical Analyses

For statistical analysis, the following programs were used: STATA version 11.0 [43] and SPSS for Windows version 17.0 [44].

We used the Kolmogorov-Smirnov's normality test. To compare the groups we used nonparametric tests, Kruskal Wallis and Mann-Whitney and Student's parametric *t*-test [45]. 

For the analyses of effect of breastfeeding and infant feeding, as well as verification of the possible factors associated with outcome, we used Pearson's Chi-square test and Fisher's Exact test. The Chi-square of linear trend was used for variables with more than two categories in which it assumed linear trend in the ratio. We also estimated the odd ratio (OR) and respective confidence intervals of 95% to associations of interest [45].

To adjust the variables we used multiple logistic regression [46] whose defined criterion for inclusion of variables was the association with the dependent variable in bivariate analysis with a *P* value lower than 0.20. For the other tests performed, the probability lower than 5% was considered as level of statistical significance (*P* < 0.05). As a quality measure of adjustment of the logistic regression models, Hosmer and Lemeshow test was used [47]. 

## 4. Results

The sample consisted of 185 children, 101 (54.6%) male and 84 (45.4%) female. The average age was 72 ± 10.7 months. Of the children in the initial sample, 52 were excluded because they were not located. The additional losses were represented by denials on the part of mothers or guardians (*n* = 3), failure to carry out all stages of the study (*n* = 12), and changes in health or use of medication that interfered with the nutritional status and body composition (*n* = 4).

Comparing the children evaluated with those who constituted the initial sample but were not included in the study (*n* = 71), no differences were found regarding sex (*P* = 0.172), mean age in months at baseline (*P* = 0.375), time of EBF (*P* = 0.197), solid food introduction age (*P* = 0.770), cow's milk consumption practice (*P* = 0.586), and infant formula (*P* = 0.576).

The median time of EBF was of 3 months and the age of introduction of solid foods was 5 months. Of the children assessed, 20.0% (*n* = 37) were not breastfed exclusively and 34.6% (*n* = 64) were breastfed for a period of 1 to 3 months and 45.4% (*n* = 84) for 4 to 6 months. With respect to the age for introducing solid food, 22.2% of the children (*n* = 41) received it previously at 3 months and 77.8% (*n* = 140) from 4 to 6 months of age. The consumption of cow's milk and infant formula occurred in 42.7% (*n* = 79) and 35.7% (*n* = 66) of cases, respectively.

Regarding nutritional status of the children, assessed by BMI index/A, we obtained the following results: 6 children (3.2%) classified in the category of thinness, 140 (75.7%) as eutrophic, 3 (1.6%) as at risk of overweight, 22 (11.9%) as overweight and 14 children (7.6%) as obese. Considering the category of overweight risk, overweight, and obesity, 21.1% of the children (*n* = 39) showed changes in nutritional status. The *z*-scores for BMI/A had an average of 0.06 ± 1.20.

According to the index H/A, a child (0.5%) had alteration, being classified of low height for the age. Evaluating by the W/A, a child (0.5%) was classified as at low weight for age, 168 (90.8%) had normal weight for age, and 16 (8.7%) had high weight for the age. 

The variables of nutritional status and body composition, with the exception of *z*-scores of BMI/A index, were not normally distributed, so the results are described as medians, as well as by the minimum and maximum values. 

 Regarding the effect of exclusive breastfeeding and infant feeding in the first six months of life in the nutritional status and body composition of children, there were no significant differences in BMI, total body fat mass, body fat mass in the android region, and waist perimeter between the different times of EBF and different ages of introduction of solid foods, as well as between children who received or not cow's milk and infant formula in the first six months of life (Table 1).

In the bivariate analyses between the variables of infant feeding and categorized parameters of nutritional status, total body fat percentage, fat percentage in the android region, and waist circumference, there were not significant associations (Table 2). There was one linear association between the EBF and the percentage of total body fat, with increase of the practice tending to increase of this percentage (Table 2).

In Tables 3, 4, and 5 are the results of the association between the confounding factors and the outcomes of interest. Among the potential confounding factors considered in relation to the nutritional status of the children, an association statistically significant was shown in the bivariate analyses in the maternal prepregnancy BMI, gestational weight gain, and sex (Table 3). Further predominance of changes in BMI/A was observed in children whose mother had prepregnancy BMI 25 kg/m^2^ (OR: 2,89; IC 95%: 1.18 to 7.09, *P* = 0.016) and had an excessive gestational weight gain (OR: 3,15; IC 95%: 1.41 to 7.06; *P* = 0.004). Female children presented lower predominance of changes of nutritional status and less change to present these changes (OR: 0,33; IC 95%: 0.15 to 0.73, *P* = 0.005) (Table 3). In addition to these variables, there were variables: included in the multivariate analyses (*P* < 0,20) birth weight (*P* = 0,136), age (*P* = 0,088) (Table 3), mother's age (*P* = 0,197), income per capita (*P* = 0,147), and hours in school (*P* = 0,097) (Table 4).

With respect to the percentage of total body fat, it was associated significantly with the changes in this parameter the maternal gestational weight gain (Table 3), daily time in active play (Table 4), and frequency of consumption of filled cookies (Table 5). Children whose mother presented excessive gestational weight gain (OR: 3,68; IC 95%: 1.50 to 9.03, *P* = 0.003) (Table 3) and children with active play time daily less than one hour (OR: 3,21; IC 95%: 1.22 to 8.41, *P* = 0.014) (Table 4) were more likely to present high percentages of total body fat. The frequency of use of filled cookies equal to or above four times a week led to a greater chance of total body fat excess compared with the consumption category of 1–3 times per week (OR: 3,75; IC 95%: 1.38 to 10.21, *P* = 0.007) (Table 5). Of the other factors evaluated as possible confounding factors, the variables of mother's schooling (*P* = 0.135) (Table 4) and consumption frequency of chocolate flavored mixes (*P* = 0.087) (Table 5) were included in the multivariate analyses. 

The gestational weight gain (Table 3), daily active play time (Table 4), and frequency in the consumption of filled cookies (Table 5) were associated in a significant way to changes in the fat percentage of the android region. Similar to that observed with regard to the percentage of total body fat, children with consumption frequency of filled cookies in the android region fat percentage were in comparison to those with intermediate consumption of these foods (OR: 3,75; IC 95%: 1.38 to 10.21, *P* = 0.007) (Table 5). Excessive weight gain during pregnancy was associated to a better chance of changing the fat percentage in the android region (OR: 2,98; IC 95%: 1,21–7,36; *P* = 0,014) (Table 3) and the time below one hour in active play also showed this result (OR: 2,55; IC 95%: 1.01 to 6.40, *P* = 0.041) (Table 4). In addition to these variables, there were the mother's age (*P* = 0.163) and time watching TV (*P* = 0.137) that included in the multivariate analyses (Table 4).

With regard to changes in waist circumference, it showed a significant association as to mother's prepregnancy BMI, gestational weight gain (Table 3) daily active play time (Table 4), and consumption frequency of filled biscuits (Table 5). Like in the other fat variables, children who had higher consumption category of filled cookies presented better chance of having high values of waist circumference in comparison to those with consumption in the intermediary category (OR: 7,26; IC 95%: 2.33 to 22.60, *P* = 0.000) (Table 5). Excessive maternal prepregnancy BMI was associated to a higher chance of change in waist circumference of children (OR: 3,36; IC 95%: 1.28 to 8.86, *P* = 0.010), the same being observed for excessive gestational weight gain (OR: 3,41; IC 95%: 1.40 to 8.27, *P* = 0.005) (Table 3). Other factors included in the multivariate analyses were the mother's age (*P* = 0.127), income per capita (*P* = 0.178) (Table 4), and percentage energy derived from lipids (*P* = 0.198) (Table 5). 

In the multivariate analyses between the exclusive breastfeeding variables and those of children feeding and the changes of the nutritional status and body composition, controlling by the confounding factors, significant independent associations were not observed for any of the analysis (Table 6).

The *P* values obtained by Hosmer and Lemeshow tests (*P* ≥ 0,05) (Table 6) showed a good adjustment of the multiple logistic regression models.

It is worth highlighting that in the multivariate analyses, some variables kept the statistical association in all the models, showing themselves as independently associated variables to the nutritional status (pregestational maternal BMI), percentage of total body fat and from the android region (maternal gestational weight gain, daily time at active play, and frequency of consumption of filled cookies), and waist circumference (pregestational maternal BMI, pregestational maternal weight gain, daily time at active play, and frequency of consumption of filled cookies), with different *P* values and odds ratio, depending on the variable of breastfeeding or child feeding evaluated in the model.

## 5. Discussion

It was observed in this study that the time of EBF was not independently associated with nutritional status, assessed as risk of overweight and obesity in children aged between 4 and 7 years. Likewise, the use of cow's milk, dairy infant formulas, and age of introduction of solid foods showed no influence on the nutritional status of these children. There were no significant associations in bivariate analyses, which did not change after adjustment by confounders. We also found no significant differences between the median values of BMI of different groups of children in times of EBF, consumption or not of cow's milk, infant dairy formulas, and ages of introduction of solid foods in infant feeding. 

Similarly, the variables of child feeding were not independently associated with total body fat percentage of children, and the values of total body fat mass did not differ between groups studied. Opposed to the initial hypothesis, there was a significant linear tendency of increasing percentage of body fat with increasing duration of EBF (*P* of linear tendency = 0.042), but this effect was attenuated after controlling by the confounders in multivariate analyses and there was no association or significant linear tendency between the variables. 

The percentage of fat in the android region and Waist circumference was measured for evaluation of localized fat in the abdominal area [32, 48]. According to the WHO, the increasing in abdominal fat in the population can provide a sensitive indicator of public health problems related to excess weight and its consequences [32] and studies show that the highest distribution of fat in this region is associated to diabetes, changes in the lipid profile and in arterial blood pressure, and risk factors to the development cardiovascular diseases [49]. In this study, the EBF time and the consumption of other foods in the first six months of life did not configure as factors associated to these parameters, with no differences in the values of android fat mass and waist circumference between the groups and with no significant statistical association with previous and subsequent changes to the control by the confounding factors. 

As demonstrated in this study, other researchers observed no significant association between breastfeeding and nutritional status and body composition. Huus et al. [23] evaluated children aged 5 years and observed that the practice of EBF held for a period less than 4 months was associated with obesity; however, in multivariate data analysis this association was not significant (OR = 1,22; IC 95%: 0,81-1,83; *P* = 0,341). 

Novaes et al. [24], in the municipality of Viçosa, MG Brazil, among children from 6 to 10 years of age, it was found that the duration of EBF was not associated with obesity (*P* = 0.713), defined as *z*-score >+2, and classified by WHO [35] after the adjustment by confounding factors related to the child and mother's characteristics. 

Novotny et al. [50], evaluating 420 American children between 6 months and 10 years of age, despite finding a significant inverse association (*P* = 0.043) between total breastfeeding and BMI of children in the analyses adjusted for confounders (birth weight, age, sex, and mother's education), report not having found association between the practice of exclusive breastfeeding and its duration with the BMI values, results are not presented in the study. 

Toschke et al. [51], assessing body composition in children 9-10 years old by DEXA, observed that the longer duration of breastfeeding was associated significantly with reduced total fat mass (*P* < 0.001), which was attenuated in 59% after adjustment by confounding factors that were socioeconomic, gestational, birth, lifestyle, and feeding. There was an inverse association between duration of breastfeeding with BMI in the bivariate analysis (*P* < 0.001) but in the adjusted model this association was not maintained (*P* = 0.238). 

Burdette et al. [52] compared children who were 5 years of age breastfed or not and found no difference in the percentage of total body fat measured by DEXA (*P* = 0.170). Breastfeeding for a time above 12 months without the use of formula did not show association with lower overweight taxes (*P* = 0.560) and, likewise, no differences were seen between the nutritional status (*P* = 0.690) and the percentage of body fat (*P* = 0.980) of the children when it was considered the introduction of solid foods before 4 months of age. The results were adjusted by confounding factors of birth and socioeconomic factors.

Kramer et al. [53] found no statistically significant differences between a group of children exclusively breastfed for longer periods in a group with relation to a group with times lower of EBF at 6.5 years of age, with regard to overweight (OR: 1,2; IC 95%: 0,8–1,6) and to the averages of the values of waist circumference (difference: 0,3 cm; IC 95%: −0.8–1.4), after adjustment for socioeconomic variables, sex, smoking during pregnancy, and birth weight. The study published later on the same sample comparing the EBF for 3 or 6 months also found the same results in relation to nutritional status and waist circumference, with no significant differences and effects of risk or protection [54].

Moorcroft et al. [55] concluded in a systematic review conducted in relation to the effect of the age of introduction of solid foods in obesity (and a portion of the studies, the excess body fat assessed by DEXA) that there is no clear association and that larger impacts relate to genetic and environmental factors.

Otherwise, the present study and the studies cited above, in which there were no associations between infant feeding practices and the outcomes studied, other studies have shown this association [20–22], are showing that they are still controversial results.

The studies evaluating the effect of breastfeeding on the nutritional status and body composition in children are mostly conducted in Western countries, as demonstrated in the discussions of this study. Thus, it is likely that differences in results are not influenced by cultural factors related to diet and lifestyle, since the populations of Western countries have similar lifestyle, to a greater or lesser degree, and have characterized a diet rich in fats and sugars and physical inactivity [56]. These factors could confound the relationship between breastfeeding and the outcomes studied, so they are used as controls in most studies.

It is observed that the studies differ on the subject as to confounding factors controlled, as how to obtain data for breastfeeding, to the type of practice measured (total or exclusive breastfeeding), and the definition used for this practice. Different anthropometric references and the cutoff points for diagnosis of the nutritional status or body composition may additionally affect the comparison of results [25, 26, 57]. 

 The absence of information on exclusive breastfeeding represents a limitation in the studies [57, 58], and in the present study we evaluated this practice. We chose to evaluate only the exclusive breastfeeding because PROLAC's followup occurs up to one year of the child's life.

It is considered that the method of obtaining the data regarding exclusive breastfeeding and feeding in the first six months of life is the main positive point of the study. We consulted the recorded data, from charts of a well structured project, with established protocol. Different results are found in studies that use recall data or that assesses breast-feeding by data obtained at the time of their practice [19, 59]. According to Adair [26], studies that recall past data on breastfeeding are subject to memory bias and discrepancies are noted between the breastfeeding analyses by data registered and data recalled.

Also as a positive point, most studies assess the effects of breastfeeding on nutritional status and total body fat; this work supplemented the assessments by parameters of fat in the abdominal region. Furthermore, the assessment of body composition was performed using DEXA, a method that has been considered the gold standard for this purpose [60].

Note also the large number and variety of confounding factors investigated that could be associated with nutritional status and body composition of children, to made a proper adjustment of the variables, could be made and sought as an independent effect of breastfeeding and infant feeding in the studied parameters. Some studies did not evaluate the confounding factors such as age, sex, birth weight, physical activity, lifestyle and current diet, socioeconomic factors, among others, which tends to undermine the analysis and discussion of results found [19, 61]. In a systematic review performed by Arenz et al. [22], it was observed that the protective effect of breastfeeding in relation to obesity was more pronounced in studies that adjusted it to less than seven potential confounders compared with those that used more than seven factors for this adjustment.

Among the confounding factors considered, there are the variables of food for the period evaluated, little considered by some researchers, evaluated in this study by two different methods. Unlike expected, it is observed that the variables from the food records and energy balance, whose determination used the average energy intake obtained by this method, were not associated with nutritional status and body composition. Errors inherent to the Food Register method, such as difficulty in describing the food, especially for quantities, may be involved in these observations [30]. 

 One factor that probably favors the divergence of the results found in the literature in relation to nutritional status is the difference in the anthropometric reference used. When it comes to assessing the nutritional status and its association with breastfeeding, we highlight differences as to the sample of the studies that have been developed for the construction of anthropometric references. The WHO reference used for evaluation of children aged under five years comes from a multicenter study and the children included were breastfed and following patterns followed satisfactory eating patterns, especially in relation to breastfeeding. This differentiates this anthropometric reference from others, which probably do not adequately express the growth of breastfed infants, especially those in EBF, since infants in the sample combine different breastfeeding practices [62]. 

Different definitions and cutoff points in relation to body composition also tend to influence the results [24, 26]. In this study, we decided to use the percentile 85 of the distribution of the sample itself by age and sex so that, just like in the evaluation of the nutritional status (when the *z*-score +1 was used as cutoff point), the risk categories were evaluated. We preferred to perform the division into percentiles within the sample itself due to the lack of national or multicentric references that included the entire age range studied.

As a limitation of this study we have to add that by prioritizing the use of data recorded of EBF and infant feeding, and because PROLAC is a program that serves a portion, but not the entire population of Viçosa, it was not possible to perform a sample that was representative or a calculation of the sample, considering the associations to be tested. To minimize this effect we included in the study all children enrolled in the program who met the inclusion criteria. An additional limitation was the losses due to failure in locating the children, because of old identification data. On the other hand, these did not affect the representativeness of the sample since it did not differ from the group analyzed. However, this is not a statistical difference between cases included and excluded in the study does not completely eliminate the risk of bias because of small sample sizes are often not sufficient to exclude type II errors. The error type II consists of not rejecting the null hypothesis when it in reality is false.

According to Dewey [57], often a small sample size is one of the factors justifying the failure to detect the effect of breastfeeding in the health parameters evaluated at later ages. Generally, large sample sizes are needed, even to be able to adjust the confounding factors [63].

A discussion held on the theme relates to publication bias: largest number of publications of studies that found positive results or with large sample sizes, which could interfere with the evaluation of the actual effect of breastfeeding on health throughout life, also interfering in the comparison between the results [25].

Importantly, the ethical issues preclude conducting controlled screening, with randomization of breastfed groups or not in studies involving human breastfeeding in humans. Thus, knowledge is obtained through observational studies with different methodologies and influence of various other factors, which helps explain some of the contradictory findings that are observed [64]. 

Excess weight and body fat are probably multifactorial and the effect of breastfeeding and feeding during the first months of life is relatively small compared to factors such as current dietary habits and living conditions and genetic factors, which makes this effect to be not quite often detected in the studies, especially those with smaller sample size [57]. 

Although not the direct targets of this work, interesting associations were found in multivariate analyses, with some variables proving to be independently associated with the parameters evaluated, demonstrating that environmental factors, in some cases even related to gestational periods, lifestyle habits, and feeding, confirmed influence on the children's health.

It is argued that, supporting the concept of nutrition transition, considering the BMI/A, children presented nearly seven times greater possibilities of alterations related to overweight (21.1%) than the deficit (3.2%). In comparison with the last national study conducted, in which the age of evaluated was range 5–9 years [5], the children of this study showed lesser prevalence of changes in nutritional status. In the cited study, 33.5% of children had values of *z*-score ≥+1 and 14,3% values of *z*-score ≥+2. 

As for feeding during the first months of life, it was observed that the practice of exclusive breastfeeding was common among the children studied; however, it can be observed, even when dealing with a program of support for breastfeeding, that there was a practice of early introduction of solid foods, as well as cow's milk, infant dairy formula in the first six months of life. The median exclusive breastfeeding was 3 months, below the level recommended by the World Health Organization, but higher than that shown in a recent study conducted in Brazilian capitals and the Federal District, which was 1.8 months [65]. It is recommended that the child gets only the mother's milk during the first six months of life and then new food be introduced (cereal, tubercles, meats, leguminous, fruit, and vegetables) slowly and gradually, in accordance with the family's meal times, at regular intervals and so as to respect the child's appetite, keeping the mother's milk up to two years of age or longer [66].

## 6. Conclusions

Unlike what has been proposed in hypotheses but consistent with some results found in the literature, exclusive breastfeeding was not confirmed as a protection against excess weight and body fat and was not associated independently to parameters of abdominal fat. The results were similar with respect to the consumption of cow's milk, dairy infant's formulas in the first six months, and the age of introduction of solid foods, without the presence of significant risk or protection.

The effects of breastfeeding on growth, development, and health of infants are indisputable, but the long-term effects in preventing cardiovascular risk factors, despite intensive discussions and a large number of publications and studies, are still controversial.

The control for the largest possible number of confounding factors, the use of reliable data on breastfeeding, appropriate definitions, and measurements of outcomes, combined with an adequate sample size, are important to reduce the existing limitations in this investigation.

## Figures and Tables

**Table 1:** Comparison of BMI, total body fat mass in the android region and waist circumference according to different practices of exclusive breastfeeding and consumption of other foods in the first 6 months of life of children from 4 to 7 years, Viçosa, MG, Brazil 2010/11.

Variables of child feeding	BMI Median (Min–Max)(kg/m^2^)	Total body fat mass Median (Min–Max) (kg)	Fat mass in android region Median (Min–Max) (g)	Waist circumference Median (Min–Max) (cm)
Months in EBF (*n* = 185)				
0 (*n* = 37)	14,90 (12,30–20,00)	2,47 (1,07–10,96)	86,00 (38,00–719,00)	54,00 (46,50–75,80)
1–3 (*n* = 64)	15,20 (12,60–21,50)	2,80 (0,90–12,34)	92,00 (41,00–71,00)	54,70 (47,10–72,30)
4–6 (*n* = 84)	15,40 (12,40–22,20)	3,50 (1,34–17,02)	123,00 (40,00–1090,00)	54,30 (47,40–83,00)
*P* value	0,394^a^	0,057^a^	0,239^a^	0,837^a^
Cow's milk (*n* = 185)				
Yes (*n* = 79)	15,20 (12,30–21,50)	2,64 (0,90–12,34)	89,00 (39,00–772,00)	54,00 (21,00–143,00)
No (*n* = 106)	15,30 (12,40–22,20)	3,25 (1,07–17,02)	115,00 (38,00–1090,00)	53,00 (28,00–162,00)
*P* value	0,469^b^	0,438^b^	0,100^b^	0,254^b^
Dairy formula (*n* = 185)				
Yes (*n* = 66)	15,00 (12,30–21,50)	3,10 (0,90–12,34)	106,00 (38,00–772,00)	55,00 (46,50–75,80)
No (*n* = 119)	15,40 (12,40–22,20)	2,92 (1,47–17,02)	99,00 (39,00–1090,00)	54,00 (47,40 −83,00)
*P* value	0,281^b^	0,726^b^	0,826^b^	0,729^b^
Age of introduction of solid foods (months) (*n* = 181)^1^				
0–3 (*n* = 41) 4–6 (*n* = 140) *P* value	15,40 (12,30–20,00) 15,20 (12,40–22,20) 0,836^b^	2,80 (0,90–10,96) 3,09 (1,07–17,02) 0,511^b^	102,00 (42,00–719,00) 105,00 (38,00–1090,0) 0,834^b^	55,00 (46,50–75,80) 54,00 (46,50–83,00) 0,464^b^

BMI: body mass index; EBF: exclusive breastfeeding; Min: minimum; Max: maximum; ^1^4 children were not included because they were being fed artificial milk and closed service at PROLAC previously to the introduction to solid foods.

^a^Kruskal Wallis's test;

^b^Mann-Whitney's test.

**Table 2:** Prevalence of BMI/A changes, percentage of total body fat, android region and waist circumference, and Crude odds ratio (confidence interval of 95%) according to different practices of exclusive breastfeeding and consumption of other foods in the first 6 months of life of children from 4 to 7 years, Viçosa, MG, Brazil, 2010/11.

Variables of child feeding	BMI/A (*z*-score)				% of body fat^1^				% of android region fat^1^				Waist circumference^1^			
	>+1 *n* (%)	≤+1 *n* (%)	Crude OR (IC 95%)	*P* value	≥P85 *n* (%)	<P85 *n* (%)	Crude OR (IC 95%)	*P* value	≥P85 *n* (%)	<P85 *n* (%)	Crude OR (IC 95%)	*P* value	≥P85 *n* (%)	<P85 *n* (%)	Crude OR (IC 95%)	*P* value
Months in EBF																
0	5 (13,5)	32 (86,5)	0,41 (0,14–1,19)	0,145	3 (8,1)	34 (91,9)	0,34 (0,09–1,27)	0,086	3 (8,1)	34 (91,9)	0,37 (0,38–1,78)	0,189	5 (13,5)	32 (86,5)	0,72 (0,24–2,15)	0,487
1–3	11 (17,2)	53 (82,8)	0,55 (0,24–1,23)		6 (9,4)	58 (90,6)	0,41 (0,15–1,10)		7 (10,9)	57 (89,1)	0,52 (0,10–1,37)		7 (10,9)	57 (89,1)	0,56 (0,21–1,48)	
4–6	23 (27,4)	61 (72,6)	1,00	0,058*	17 (20,2)	67 (79,8)	1,00	0,042*	16 (19,0)	68 (81,0)	1,00	0,079*	15 (17,9)	69 (82,1)	1,00	
Cow's milk																
Yes	15 (19,0)	64 (81,0)	0,80 (0,38–1,65)	0,547	8 (10,1)	71 (89,9)	0,55 (0,22–1,23)	0,185	9 (11,4)	79 (88,6)	0,67 (0,28–1,60)	0,369	11 (13,9)	68 (86,1)	0,71 (0,24–2,10)	0,824
No	24 (22,6)	82 (77,4)	1,00		18 (17,0)	88 (83,0)	1,00		17 (16,0)	89 (84,0)	1,00		16 (15,1)	90 (84,9)	1,00	
Infant dairy formulas																
Yes	11 (16,7)	55 (83,3)	0,65 (0,30–1,41)	0,273	9 (13,6)	57 (86,4)	0,95 (0,39–2,26)	0,903	12 (18,2)	54 (81,8)	1,66 (0,72–3,85)	0,229	11 (16,7)	55 (83,3)	1,28 (0,56–2,97)	0,552
No	28 (23,5)	91 (76,5)	1,00		17 (14,3)	102 (85,7)	1,00		14 (11,8)	105 (88,2)	1,00		16 (13,4)	103 (86,6)	1,00	
Age of introduction of solid foods (months)^2^																
0–3	10 (24,4)	31 (75,6)	1,29 (0,56–2,94)	0,544	3 (7,3)	38 (92,7)	0,42 (0,12–1,49)	0,207**	4 (9,8)	37 (90,2)	0,61 (0,20–1,89)	0,392**	5 (12,2)	36 (87,8)	0,78 (0,28–2,23)	0,652
4–6	28 (20,0)	112 (80,0)	1,00		22 (15,7)	118 (84,3)	1,00		21 (15,0)	119 (85,0)	1,00		67 (15,0)	73 (85,0)	1,00	

BMI: body mass index; A: age; EBF: exclusive breastfeeding; OR: odds ratio*; *IC: interval of confidence; *P* values derived from Chi-square test, of linear tendency* and Fisher's Exact** and*; ^1^percentiles calculated from among sample children by sex and age;^2^4 children were not included because they were being fed artificial milk and closed service at PROLAC previously to the introduction to solid foods.

**Table 3:** Prevalence of BMI/A changes, percentage of total body fat, from android region and waist circumference, and Crude odds ratio (confidence interval of 95%) according to maternal, pregnancy and birth variables, sex, age, and occurrence of hospitalizations of children from 4 to 7 years of age, Viçosa, MG, Brazil, 2010/11.

Variables	BMI/A (*z*-score)				% of body fat^1^				% of fat in android region^1^				Waist circumference^1^			
	>+1 *n* (%)	≤+1 *n* (%)	OR (IC 95%)	*P* value	≥P85 *n* (%)	<P85 *n* (%)	OR (IC 95%)	*P* value	≥P85 *n* (%)	<P85 *n* (%)	OR (IC 95%)	*P* value	≥P85 *n* (%)	<P85 *n* (%)	OR (IC 95%)	*P* value
Pregestational BMI (kg/m^2^)^2^																
<24,99	29 (18,7)	126 (81,3)	1,00	**0,016**	23 (14,8)	132 (85,2)	1,00	1,000*	23 (14,8)	132 (85,2)	1,00	1,000*	19 (12,3)	136 (87,7)	1,00	**0,010**
≥25,00	10 (40,0)	15 (60,0)	2,89 (1,18–7,09)		3 (12,0)	22 (88,0)	0,78 (0,21–2,82)		3 (12,0)	22 (88,0)	0,78 (0,21–2,82)		8 (32,0)	17 (68,0)	3,36 (1,28–8,86)	
Gestational weight gain^3^																
Not excessive	22 (16,2)	114 (83,8)	1,00	**0,004**	14 (10,3)	122 (89,7)	1,00	**0,003**	15 (11,0)	121 (89,0)	1,00	**0,014**	15 (11,0)	121 (89,0)	1,00	**0,005**
Excessive	14 (37,8)	23 (62,2)	3,15 (1,41–7,06)		11 (29,7)	26 (70,3)	3,68 (1,50–9,03)		10 (27,0)	27 (73,0)	2,98 (1,21–7,36)		11 (29,7)	26 (70,3)	3,41 (1,40–8,27)	
Mother's smoking during pregnancy^4^																
No	34 (19,9)	137 (80,1)	1,00	0,275*	25 (14,6)	146 (85,4)	1,00	1,000*	25 (14,6)	146 (85,4)	1,00	1,000*	25 (14,6)	146 (85,4)	1,00	0,692*
Yes	4 (33,3)	8 (66,7)	2,01 (0,57–7,08)		1 (8,3)	11 (91,7)	0,53 (0,06–4,29)		1 (8,3)	11 (91,7)	0,53 (0,06–4,29)		2 (16,7)	10 (83,3)	1,16 (0,24–5,65)	
Birth weight (g)																
2500–2999	8 (12,9)	54 (87,1)	1,00	**0,136**	5 (8,1)	57 (91,9)	1,00	0,248	7 (11,3)	55 (88,7)	1,00	0,737	7 (11,3)	55 (88,7)	1,00	
3000–3499	17 (27,0)	46 (73,0)	2,49 (0,98–6,31)		11 (17,5)	52 (82,5)	2,41 (0,78–7,40)		10 (15,9)	53 (84,1)	1,48 (0,52–4,18)		10 (15,9)	53 (84,1)	1,48 (0,52–4,18)	0,660 0,400**
3500–3999	14 (23,1)	46 (76,9)	2,05 (0,79–5,33)		10 (16,7)	50 (83,3)	2,28 (0,73–7,12)		9 (15,0)	51 (85,0)	1,38 (0,48–3,99)		10 (16,7)	50 (83,3)	1,57 (0,55–4,42)	
Sex																
Male	29 (28,7)	72 (71,3)	1,00	**0,005**	14 (13,9)	87 (86,1)	1,00	0,934	14 (13,9)	87 (86,1)	1,00	0,934	14 (13,9)	87 (86,1)	1,00	0,757
Female	10 (11,9)	74 (88,1)	0,33 (0,15–0,73)		12 (14,3)	72 (85,7)	1,03 (0,45–2,38)		12 (14,3)	72 (85,7)	1,03 (0,45–2,38)		13 (15,5)	71 (84,5)	1,14 (0,50–2,57)	
Age (years)																
4-5	13 (15,5)	71 (84,5)	1,00	**0,088**	12 (14,3)	72 (85,7)	1,00	0,934	12 (14,3)	72 (85,7)	1,00	0,934	13 (15,5)	71 (84,5)	1,00	0,757
6-7	26 (25,7)	75 (74,3)	1,89 (0,90–3,97)		14 (13,9)	87 (86,1)	0,96 (0,42–2,21)		14 (13,9)	87 (86,1)	0,96 (0,42–2,21)		14 (13,9)	87 (86,1)	0,88 (0,38–1,99)	
Hospitalizations																
Yes	14 (18,9)	60 (81,1)	1,00	0,556	10 (13,5)	64 (86,5)	1,00	0,863	10 (13,5)	64 (86,5)	1,00	0,863	10 (13,5)	64 (86,5)	1,00	0,734
No	25 (22,5)	86 (77,5)	1,24 (0,59–2,59)		16 (14,4)	95 (85,6)	1,08 (0,46–2,52)		16 (14,4)	95 (85,6)	1,08 (0,46–2,52)		17 (15,3)	94 (84,7)	1,15 (0,49–2,69)	

BMI: body mass index; A: age; OR: odds ratio*; *IC: interval of confidence; *P* values derived from Chi-square test, Fisher's Exact* and of linear tendency**; ^1^percentiles calculated from among sample children by sex and age; ^2^ *n* = 180; ^3^ *n* = 173; ^4^ *n* = 183. Values in bold represent statistical significance for inclusion in the multivariate analysis (*P*< 0,20).

**Table 4:** Prevalence of BMI/A change, percentage of total body fat, android region and waist circumference, and Crude odds ratio (confidence interval of 95%) according to sociodemographic and lifestyle habits of children from 4 to 7 years of age, Viçosa, MG, Brazil, 2010/11.

Variables	BMI/A (*z*-score)				% of body fat^1^				% of fat in the android region^1^				Waist circumference^1^			
	>+1 *n* (%)	≤+1 *n* (%)	OR (IC 95%)	*P* value	≥P85 *n* (%)	<P85 *n* (%)	OR (IC 95%)	*P* value	≥P85 *n* (%)	<P85 *n* (%)	OR (IC 95%)	*P* value	≥P85 *n* (%)	<P85 *n* (%)	OR (IC 95%)	*P* value
Mother's age (yrs)^2^																
20–28	17 (25,4)	50 (74,6)	1,00	**0,197**	13 (19,4)	54 (80,6)	1,00	0,242	11 (16,4)	56 (83,6)	1,00	**0,163**	8 (11,9)	59 (88,1)	1,00	**0,127**
29–34	9 (13,8)	56 (86,2)	0,47 (0,19–1,15)		6 (9,2)	59 (90,8)	0,42 (0,15–1,19)		5 (7,7)	60 (92,3)	0,42 (0,14–1,30)		7 (10,8)	58 (89,2)	0,89 (0,30–2,61)	
35–51	13 (25,0)	39 (75,0)	0,98 (0,42–2,26)		7 (13,5)	45 (86,5)	0,64 (0,24–1,75)		10 (19,2)	42 (80,8)	1,21 (0,47–3,12)		12 (23,1)	40 (76,9)	2,21 (0,83–5,89)	
Mother's schooling (yrs)^2^																
>8	22 (21,6)	80 (78,4)	1,00	0,924	18 (17,6)	84 (82,4)	1,00	**0,135**	16 (15,7)	86 (84,3)	1,00	0,520	16 (15,7)	86 (84,6)	1,00	0,690
≤8	17 (21,0)	64 (79,0)	0,96 (0,47–1,97)		8 (9,9)	73 (90,1)	0,51 (0,21–1,24)		10 (12,3)	71 (87,7)	0,76 (0,32–1,77)		11 (13,6)	70 (86,4)	0,84 (0,36–1,93)	
Income *per capita* (reais)^3^																
40,57–204,00	10 (15,6)	54 (84,4)	1,00	0,345	9 (14,1)	55 (85,9)	1,00	0,975	9 (14,1)	55 (85,9)	1,00	0,446	8 (12,5)	56 (87,5)	1,00	**0,178**
204,37–350,0	13 (21,7)	47 (78,3)	1,49 (0,60–3,72)		8 (13,3)	52 (86,7)	0,94 (0,33–2,62)		6 (10,0)	54 (90,0)	0,68 (0,22–2,03)		6 (10,0)	54 (90,0)	0,78 (0,25–2,40)	
357,0–3333,33	16 (26,2)	45 (73,8)	1,92 (0,79–4,64)	0,147*	9 (14,8)	52 (85,2)	1,06 (0,39–2,87)		11 (18,0)	50 (82,0)	1,34 (0,51–3,51)		13 (21,3)	48 (78,7)	1,89 (0,72–4,95)	
Residence																
Rural	4 (28,6)	10 (71,4)	1,00	0,498**	2 (14,3)	12 (85,7)	1,00	1,000**	0 (0,0)	14 (100,0)	1,00	0,224	1 (7,1)	13 (92,9)	1,00	0,697**
Urban	35 (20,5)	136 (79,5)	0,64 (0,19–2,17)		24 (14,0)	147 (86,0)	0,98 (0,20–4,65)		26 (94,4)	145 (90,5)	—		26 (15,2)	145 (84,8)	2,33 (0,29–18,59)	
Time TV (hours)																
≤2	16 (18,0)	73 (82,0)	1,00	0,319	11 (12,4)	78 (87,6)	1,00	0,523	9 (10,1)	80 (89,9)	1,00	**0,137**	11 (12,4)	78 (87,6)	1,00	0,407
>2	23 (24,0)	73 (76,0)	1,44 (0,70–2,93)		15 (15,6)	81 (84,4)	1,31 (0,56–3,03)		17 (17,7)	79 (82,3)	1,91 (0,80–4,55)		16 (16,7)	80 (83,3)	1,42 (0,62–3,24)	
Hours in school^4^																
>4	25 (25,5)	73 (74,5)	1,00	**0,097**	13 (13,3)	85 (86,7)	1,00	0,671	13 (13,3)	85 (86,7)	1,00	0,671	14 (14,3)	84 (85,7)	1,00	0,822
≤4	13 (15,5)	71 (84,5)	0,53 (0,25–1,12)		13 (15,5)	71 (84,5)	1,20 (0,52–2,75)		13 (15,5)	71 (84,5)	1,20 (0,52–2,75)		13 (15,5)	71 (84,5)	1,09 (0,48–2,49)	
Time in active play (hours)^5^																
>1	15 (17,9)	69 (82,1)	1,00	0,327	6 (7,1)	78 (92,9)	1,00	**0,014**	7 (8,3)	77 (91,7)	1,00	**0,041**	6 (7,1)	78 (92,9)	1,00	**0,009**
≤1	24 (23,8)	77 (76,2)	1,43 (0,69–2,95)		20 (19,8)	81 (80,2)	3,21 (1,22–8,41)		19 (18,8)	82 (81,2)	2,55 (1,01–6,40)		21 (20,8)	80 (79,2)	3,41 (1,31–8,90)	
Time in light activities (hours)^6^																
≤1	27 (24,1)	85 (75,9)	1,00	0,211	13 (11,6)	99 (88,4)	1,00	0,236	13 (11,6)	99 (88,4)	1,00	0,236	15 (13,4)	97 (86,6)	1,00	0,566
>1	12 (16,4)	61 (83,6)	0,62 (0,29–1,32)		13 (17,8)	60 (82,2)	1,65 (0,71–3,79)		13 (17,8)	60 (82,2)	1,65 (0,71–3,79)		12 (16,4)	61 (83,6)	1,27 (0,56–2,90)	
Physical education^4^																
Yes	22 (19,5)	91 (80,5)	1,00	0,712	19 (16,8)	94 (83,2)	1,00	0,212	19 (16,8)	94 (83,2)	1,00	0,212	19 (16,8)	94 (83,2)	1,00	0,336
No	15 (21,7)	54 (78,3)	1,14 (0,55–2,40)		7 (10,1)	62 (89,9)	0,56 (0,22–1,40)		7 (10,1)	62 (89,9)	0,56 (0,22–1,40)		8 (11,6)	61 (88,4)	0,65 (0,26–1,57)	

BMI: body mass index;A: age; TV: television; OR: odds ratio; IC: interval of confidence; *P* values derived from Chi-square test, of linear tendency* and Fisher's Exact**; ^1^percentiles calculated between children from the sample by sex and age;^ 2^ *n* = 184; ^3^values categorized by tertiles; ^4^ *n* = 182; ^5^riding a bike, play with a Ball, run, among others;^ 6^trolley, doll house, house, among others, or doing home work; values in bold represent statistical significance for inclusion in the multivariate analyses (*P*< 0,20).

**Table 5:** Prevalence of BMI/A changes, percentage of total body fat, from the android region and waist circumference, and Crude odds ratio (interval of confidence of 95%) in accordance with feeding variables of children from 4 to 7 years, Viçosa, MG, Brazil, 2010/11.

Variables	BMI/A (*z*-score)				% of body fat^1^				% of fat of android region^1^				Waist circumference^1^			
	>+1 *n* (%)	≤+1 *n* (%)	OR (IC 95%)	*P* value	≥P85 *n* (%)	<P85 *n* (%)	OR (IC 95%)	*P* value	≥P85 *n* (%)	<P85 *n* (%)	OR (IC 95%)	*P* value	≥P85 *n* (%)	<P85 *n* (%)	OR (IC 95%)	*P* value
Energy derived from carbohydrates (%)																
≤65	38 (21,8)	136 (78,2)	1,00	0,463*	25 (14,4)	149 (85,6)	1,00	1,000*	25 (14,4)	149 (85,6)	1,00	1,000*	27 (15,5)	147 (84,5)	1,00	0,372*
>65	1 (9,1)	10 (90,9)	0,36 (0,04–2,88)		1 (9,1)	10 (90,9)	0,59 (0,07–4,86)		1 (9,1)	10 (90,9)	0,59 (0,07–4,86)		0 (0,0)	11 (100,0)	—	
Energy derived from lipids (%)																
≤35	34 (22,2)	119 (77,8)	1,00	0,405	20 (13,1)	133 (86,9)	1,00	0,401	20 (13,1)	133 (86,9)	1,00	0,401	20 (13,1)	133 (86,9)	1,00	**0,198**
>35	5 (15,6)	27 (84,4)	0,65 (0,23–1,81)		6 (18,8)	26 (81,2)	1,53 (0,56–4,19)		6 (18,8)	26 (81,2)	1,53 (0,56–4,19)		7 (21,9)	25 (78,1)	1,86 (0,71–4,86)	
Energy balance																
Nonpositive	28 (21,7)	101 (78,3)	1,00	0,752	19 (14,7)	110 (85,3)	1,00	0,689	20 (15,5)	109 (84,5)	1,00	0,389	21 (16,3)	108 (83,7)	1,00	0,325
Positive	11 (19,6)	45 (80,4)	0,88 (0,40–1,93)		7 (12,5)	49 (87,5)	0,83 (0,32–2,09)		6 (10,7)	50 (89,3)	0,65 (0,24–1,73)		6 (10,7)	50 (89,3)	0,62 (0,23–1,62)	
Frequency candy, lollipops, gums																
<1 day/week	6 (28,6)	15 (71,4)	1,00		5 (23,8)	16 (76,2)	1,00	0,361	2 (9,5)	19 (90,5)	1,00	0,787	5 (23,8)	16 (76,2)	1,00	0,434
1–3 days/week	15 (21,1)	56 (78,9)	0,67 (0,23–2,02)	0,646	10 (14,1)	61 (85,9)	0,52 (0,15–1,75)		11 (15,5)	60 (84,5)	1,74 (0,35–8,56)		9 (12,7)	62 (87,3)	0,46 (0,13–1,58)	
≥4 days/week	18 (19,4)	75 (80,6)	0,60 (0,20–1,76)	0,402**	11 (11,8)	82 (88,2)	0,43 (0,13–1,40)	0,202**	13 (14,0)	80 (86,0)	1,54 (0,32–7,42)		13 (14,0)	80 (86,0)	0,52 (0,16–1,66)	
Frequency filled cookies																
<1 day/week	7 (21,2)	26 (78,8)	1,00	0,428	4 (12,1)	29 (87,9)	1,00		4 (12,1)	29 (87,9)	1,00	0,022***	4 (12,1)	29 (87,9)	1,00	0,000****
1–3 days/week	14 (17,1)	68 (82,9)	0,76 (0,27–2,10)		6 (7,3)	76 (92,7)	0,57 (0,15–2,17)	0,022***	6 (7,3)	76 (92,7)	0,57 (0,15–2,17)		4 (4,9)	78 (95,1)	0,37 (0,08–1,58)	
≥4 days/week	18 (25,7)	52 (74,3)	1,28 (0,47–3,46)		16 (22,9)	54 (77,1)	2,14 (0,65–7,02)		16 (22,9)	54 (77,1)	2,14 (0,65–7,02)		19 (27,1)	51 (72,9)	2,70 (0,84–8,70)	
Frequency chocolate milk																
<1 day/week	10 (17,9)	46 (82,1)	1,00		5 (8,9)	51 (91,1)	1,00		6 (10,7)	50 (89,3)	1,00	0,337	6 (10,7)	50 (89,3)	1,00	0,348
1–3 days/week	6 (28,6)	15 (71,4)	1,84 (0,57–5,92)	0,588	6 (28,6)	15 (71,4)	4,08 (1,09–15,26)	**0,087**	5 (23,8)	16 (76,2)	2,60 (0,70–9,68)		5 (23,8)	16 (76,2)	2,60 (0,70–9,68)	
≥4 days/week	23 (21,3)	85 (78,7)	1,24 (0,55–2,84)		15 (13,9)	93 (86,1)	1,64 (0,56–4,78)		15 (13,9)	93 (86,1)	1,34 (0,49–3,68)		16 (14,8)	92 (85,2)	1,45 (0,53–3,94)	
Frequency fried foods																
<1 day/week	5 (20,0)	20 (80,0)	1,00	0,858	4 (16,0)	21 (84,0)	1,00	0,355	4 (16,0)	21 (84,0)	1,00	0,355	4 (16,0)	21 (84,0)	1,00	0,540
1–3 days/week	23 (22,5)	79 (77,5)	1,16 (0,39–3,44)		17 (16,7)	85 (83,3)	1,05 (0,32–3,45)		17 (16,7)	85 (83,3)	1,05 (0,32–3,45)		17 (16,7)	85 (83,3)	1,05 (0,32–3,45)	
≥4 days/week	11 (19,0)	47 (81,0)	0,94 (0,28–3,04)		5 (8,6)	53 (91,4)	0,49 (0,12–2,03)		5 (8,6)	53 (91,4)	0,49 (0,12–2,03)		6 (10,3)	52 (89,7)	0,60 (0,15–2,36)	
Frequency soft drinks																
<1 day/week	9 (21,4)	33 (78,6)	1,00	0,476	5 (11,9)	37 (88,1)	1,00	0,624	6 (14,3)	36 (85,7)	1,00	0,635	8 (19,0)	34 (81,0)	1,00	0,614
1–3 days/week	24 (19,4)	100 (80,6)	0,88 (0,37–2,08)		17 (13,7)	107 (86,3)	0,95 (0,35–2,60)		16 (12,9)	108 (87,1)	0,89 (0,32–2,44)		16 (12,9)	108 (87,1)	0,63 (0,25–1,60)	
≥4 days/week	6 (31,6)	13 (68,4)	1,69 (0,50–5,71)		4 (21,1)	15 (78,9)	1,12 (0,25–5,07)	0,401**	4 (21,1)	15 (78,9)	1,60 (0,39–6,50)		3 (15,8)	16 (84,2)	0,80 (0,18–3,41)	

BMI: body mass index; A: age; EBF: exclusive breastfeeding materno; OR: odds ratio*; *IC: interval of confidence; ^1^percentiles calculated between children from the sample by sex and age; *P* values derived from Chi-square test; Fisher's Exact* and linear tendency**; OR significant between ≥4 days/week with relation to a 1–3 days/week (OR: 3,75; IC 95%: 1,38–10,21)***; OR significant between ≥4 days/week in relation to 1–3 days/week (OR: 7,26; IC 95%: 2,33–22,60)****; values in bold represent statistical significance to include in the multivariate analyses (*P*< 0,20).

**Table 6:** Values of Crude and adjusted odds ratio (intervals of confidence of 95%) in alternations of BMI/A percentage of body fat, total, and from the android region and waist circumference in accordance with different exclusive breastfeeding practices and consumption of other foods in the first 6 months of life of children from 4 to 7 years, Viçosa, MG, Brazil, 2010/11.

Variables of child feeding	BMI/I (*z*-score)^1^				% of body fat^1^				% of fat from the android region^2^				Waist circumference^2^			
	OR Crude (IC 95%)	OR adjusted^3^ (IC 95%)	*P* value^3^	*P* value*	OR Crude (IC 95%)	OR adjusted^4^ (IC 95%)	*P* value^4^	*P* value*	OR Crude (IC 95%)	OR adjusted^5^ (IC 95%)	*P* value^5^	*P* value*	OR Crude (IC 95%)	OR adjusted^6^ (IC 95%)	*P* value^6^	*P* value*
Months in EBF																
0	0,41 (0,14–1,19)	0,34 (0,09–1,26)	0,192	0,485	0,34 (0,09–1,27)	0,39 (0,08–1,74)	0,280	0,650	0,37 (0,38–1,78)	0,56 (0,13–2,30)	0,576	0,727	0,72 (0,24–2,15)	0,79 (0,18–3,40)	0,902	0,704
1–3	0,55 (0,24–1,23)	0,52 (0,19–1,37)			0,41 (0,15–1,10)	0,44 (0,13–1,43)			0,52 (0,10–1,37)	0,63 (0,22–1,81)			0,56 (0,21–1,48)	0,77 (0,23–2,57)		
4–6	1,00	1,00	0,124**		1,00	1,00	0,077**		1,00	1,00	0,139**		1,00	1,00		
Cow's milk																
Yes	0,80 (0,38–1,65)	0,67 (0,27–1,64)	0,383	0,750	0,55 (0,22–1,23)	0,47 (0,16–1,38)	0,171	0,720	0,67 (0,28–1,60)	0,58 (0,22–1,55)	0,279	0,472	0,91 (0,39–2,08)	0,76 (0,31–2,40)	0,780	0,383
No	1,00	1,00			1,00	1,00			1,00	1,00			1,00	1,00		
Infant dairy formulas																
Yes	0,65 (0,30–1,41)	0,83 (0,33–2,07)	0,697	0,613	0,95 (0,39–2,26)	0,88 (0,31–2,50)	0,813	0,859	1,66 (0,72–3,85)	1,77 (0,68–4,64)	0,241	0,210	1,28 (0,56–2,97)	1,61 (0,52–4,98)	0,409	0,185
No	1,00	1,00			1,00	1,00			1,00	1,00			1,00	1,00		
Age of introduction of solid foods (months)																
0–3	1,29 (0,56–2,94)	1,46 (0,53–4,04)	0,460	0,225	0,42 (0,12–1,49)	0,47 (0,12–1,87)	0,285	0,473	0,61 (0,20–1,89)	0,68 (0,18–2,48)	0,557	0,214	0,78 (0,28–2,23)	0,63 (0,16–2,46)	0,508	0,080
4–6	1,00	1,00			1,00	1,00			1,00	1,00			1,00	1,00		

BMI: body mass index; A: age; EBF: exclusive breastfeeding; OR: odds ratio; IC: interval of confidence; ^1^cutoff considered for change inscore-*z*of BMI/A > +1; ^2^cutoff considered for change: percentile: ≥85, of the percentiles calculated among children of the sample by sex and age; *P* values derived from Hosmer's and Lemeshow's test*; linear tendency**; ^3^adjustment by prégestational BMI, gestational weight gain, birth weight, sex, age at the moment of assessment, mother's age, income per capita, and daily hours at school; ^4^adjustment per gestational weight gain, mother's education, daily time at active play, frequency of consumption of filled cookies, and chocolate milk; ^5^adjustment per gestational weight gain, mother's age, daily time watching TV, daily time at active play, and frequency of consumption of filled cookies; ^6^adjustment per prégestational BMI, gestational weight gain, mother's age, income per capita, daily time at active play, energy derived from lipids, and frequency of consumption of filled cookies.
